# Effects of Low Carbohydrate High Protein (LCHP) diet on atherosclerotic plaque phenotype in ApoE/LDLR^−/−^ mice: FT-IR and Raman imaging

**DOI:** 10.1038/srep14002

**Published:** 2015-09-22

**Authors:** T. P. Wrobel, K. M. Marzec, S. Chlopicki, E. Maślak, A. Jasztal, M. Franczyk-Żarów, I. Czyżyńska-Cichoń, T. Moszkowski, R. B. Kostogrys, M. Baranska

**Affiliations:** 1 Faculty of Chemistry, Jagiellonian University, ul. Ingardena 3, 30-060 Krakow, Poland; 2 Jagiellonian Centre for Experimental Therapeutics (JCET), Jagiellonian University, ul. Bobrzynskiego 14, 30-348 Krakow, Poland; 3 Department of Experimental Pharamacology, Chair of Pharmacology, Jagiellonian University Medical College, ul. Grzegorzecka 16, 31-531 Krakow, Poland; 4 Department of Human Nutrition, Faculty of Food Technology, Agricultural University of Kraków, ul.Balicka 122, 30-149 Krakow, Poland; 5 Department of Automatics and Bioengineering, AGH University of Science and Technology Krakow, Al. Mickiewicza 30, 30-059 Krakow, Poland

## Abstract

Low Carbohydrate High Protein (LCHP) diet displays pro-atherogenic effects, however, the exact mechanisms involved are still unclear. Here, with the use of vibrational imaging, such as Fourier transform infrared (FT-IR) and Raman (RS) spectroscopies, we characterize biochemical content of plaques in Brachiocephalic Arteries (BCA) from ApoE/LDLR^−/−^ mice fed LCHP diet as compared to control, recomended by American Institute of Nutrition, AIN diet. FT-IR images were taken from 6–10 sections of BCA from each mice and were complemented with RS measurements with higher spatial resolution of chosen areas of plaque sections. In aortic plaques from LCHP fed ApoE/LDLR^−/−^ mice, the content of cholesterol and cholesterol esters was increased, while that of proteins was decreased as evidenced by global FT-IR analysis. High resolution imaging by RS identified necrotic core/foam cells, lipids (including cholesterol crystals), calcium mineralization and fibrous cap. The decreased relative thickness of the outer fibrous cap and the presence of buried caps were prominent features of the plaques in ApoE/LDLR^−/−^ mice fed LCHP diet. In conclusion, FT-IR and Raman-based imaging provided a complementary insight into the biochemical composition of the plaque suggesting that LCHP diet increased plaque cholesterol and cholesterol esters contents of atherosclerotic plaque, supporting the cholesterol-driven pathogenesis of LCHP–induced atherogenesis.

Methods of obtaining spatially resolved information about chemical composition of samples have developed significantly over the recent years also in the form of infrared (IR) and Raman imaging^1^. In the past, both FT-IR^2^,^3^,^4^ and Raman^5^,^6^,^7^ spectroscopy were successfully applied for studying chemical composition of the atherosclerotic plaque – a more thorough review is available in the literature^8^. The techniques were usually used separately, however, there are exceptions to this trend^9^,^10^ as well as the work presented here. We have previously studied the lipid composition^11^ and protein profile^12^ of atherosclerotic plaques, as well as the progression of the disease along with quantification of the plaque’s area^13^. Moreover, a methodology combining three techniques of FT-IR, Raman and Atomic Force Microscopy (AFM) imaging with later proper histologic staining of the same tissue fragment was developed, which allowed maximum of information to be obtained^10^.

Recent evidence suggests that Low Carbohydrate High Protein (LCHP) diet displays pro-atherogenic effects even more pronounced than that of the Western diet^14^, but the mechanisms involved are still unclear. Foo *et al.*^15^ suggested that this phenomenon was due to substantially reduced number of bone marrow and peripheral blood endothelial progenitor cells (EPCs), and impaired vascular regenerative capacity. Interestingly, EPCs from mice on a LCHP diet manifested lower levels of activated (phosphorylated) Akt, a serine-threonine kinase important in EPC mobilization, proliferation and survival, resulting in a reduced ischemia-induced neovascularization in LCHP diet fed ApoE^−/−^ mice. In a previous work, Kostogrys *et al.*^14^ observed that the area of atherosclerotic plaques in aortic roots or BCA from LCHP diet fed mice was substantially increased, as compared to mice fed control diet, and was characterized by increased lipids and cholesterol contents, macrophage infiltration (MOMA-2) and activity of MMPs (zymography). It was shown, that pro-atherogenic phenotype of LCHP fed ApoE/LDLR^−/−^ mice was associated with increased plasma concentration of total cholesterol, LDL and VLDL cholesterol content, as well as increased triacylglycerols content in VLDL. These findings suggested that LCHP diet induced atherogenesis in ApoE/LDLR^−/−^ mice was associated with cholesterol-driven mechanisms but other mechanisms could not be excluded^16^.

The aim of the present work was to take advantage of vibrational microspectroscopy such as FT-IR and Raman and to characterize biochemical contents of atherosclerotic plaques in BCA from ApoE/LDLR^−/−^ mice fed LCHP diet. In particular we aim to confirm cholesterol-driven mechanisms of LCHP-induced atherogenesis by directly measuring cholesterol content of the plaques.

## Results

### Quantitative assessment with FT-IR imaging – global changes

**Figure 1:**
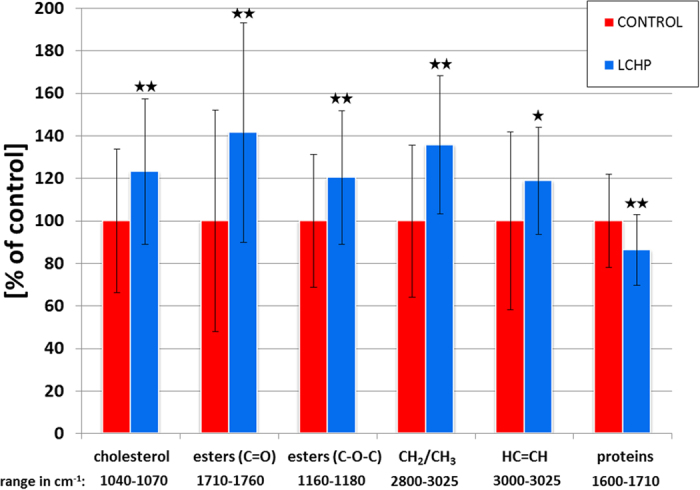
Quantification results of the most important band changes induced by LCHP diet. Data scaled to the mean value of AIN (control) group; error bars are standard deviations; *p < 0.05, **p < 0.01 for Mann-Whitney-Wilcoxon (MWW) test.

The differences in plaque composition for the main components in LCHP and control (recomended by American Institute of Nutrition-AIN diet) groups analyzed in the most important spectroscopic ranges are shown in Fig. 1. AIN is a standardized diet and serves as a control “normal” diet. LCHP diet and AIN diet groups consisted of 5 and 6 animals, respectively, with 6–10 sections per animal giving the final dataset equal to 41 (LCHP) and 53 (AIN) sections. The ranges chosen for quantification are based on previous works performed on pure substances as well as on tissue sections^8^,^11^.

A general decrease in protein content of 15% (**p < 0.01) was observed for all protein bands in the LCHP group. With regards to lipids, statistically significant increase in both cholesterol and in esters content was observed. Cholesterol increased by 23%, while the carbonyl band indicated an increase in esters content of up to 41%. However, this band’s intensity is potentially strongly influenced by scattering effects, therefore, a second band at around 1170 cm^−1^ was used. This band has a lower standard deviation and indicates an increase of only 20% for esters. This remains in accordance with the increase of CH_2_/CH_3_ band of around 36%, which should be the sum of the increases from cholesterol and from esters. Moreover, the signal of double C = C bonds also increased by almost 19% - this suggests that the increase of esters occurred mostly by an accumulation of unsaturated esters. The observed changes all have high statistical significance (p < 0.01) with the exception of C = C signal, which unfortunately has higher standard deviation, due to a small range of integration (3000–3025 cm^−1^) and higher susceptibility to noise.

Calcification was also observed in BCAs obtained from mice on AIN (3 animals) and LCHP (3 animals) diet. Some examples of calcification smaller than 2 μm in diameter were observed with Raman mapping (Figure S2).

### Quantitative assessment of local plaque composition with FT-IR imaging

**Figure 2:**
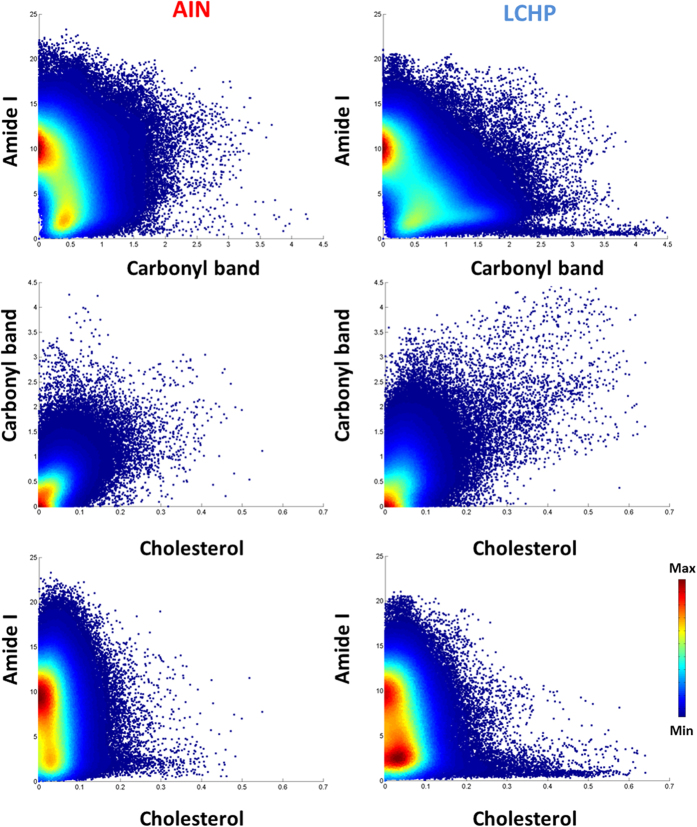
Scatter plots of individual pixels from AIN (control) and LCHP groups, based on integration of protein, ester and cholesterol signals. Each point corresponds to an area roughly of 5.5 × 5.5 micrometers^2^ and the values on the axes represent band intensity.

Scatter plots with integrations from individual pixels from all images offer an easy insight into colocalization of chemical species and are presented in Fig. 2. Each point corresponds to a single pixel from an FT-IR image.

Since the number of points is quite large (>280000) a density based scatter plot was chosen to properly demonstrate the relationships in the data rather than a normal scatter plot. The scatter plot between protein signal (Amide I band) and the ester signal (Carbonyl band) shows a group of pixels of very high ester content, while having very little protein content in the LCHP group – lying very close to the x axis. This indicates formation of areas (lipid cores) within the plaques of mice fed LCHP diet – containing very little proteins, while having significant amounts of esters. Moreover, there is an increase in the density of pixels between units 1 and 1.5 of the carbonyl integration, showing that apart from forming extremely lipid-rich regions, the esters are mostly increasing in regions of very small Amide I integration between 1 and 5 units. The second pair of scatter plots shows the co-occurrence of esters and cholesterol in the atherosclerotic plaques. Interestingly, there are no groups of pixels in LCHP group lying close to either of the axes with high content of either of the lipid groups. This suggests that there are very few areas of extremely high content of only esters or only cholesterol – in high contents they are colocalized. The third pair shows complementary information of cholesterol and protein relation and produces a very similar image as the previous ones. The only difference is seen in comparing it with the ester and protein plots, from which it can be observed that esters mix with proteins more easily than cholesterol, since the central part of the scatter plot is not being occupied. Moreover, on this plot there also is a group of pixels located close to the x axis having very high cholesterol content with almost no protein. It is those pixels that occupy the central part of high esters and high cholesterol plot, clearly indicating that these regions are almost purely lipids without protein component.

### Qualitative assessment of plaque composition with Raman and FT-IR imaging

**Figure 3:**
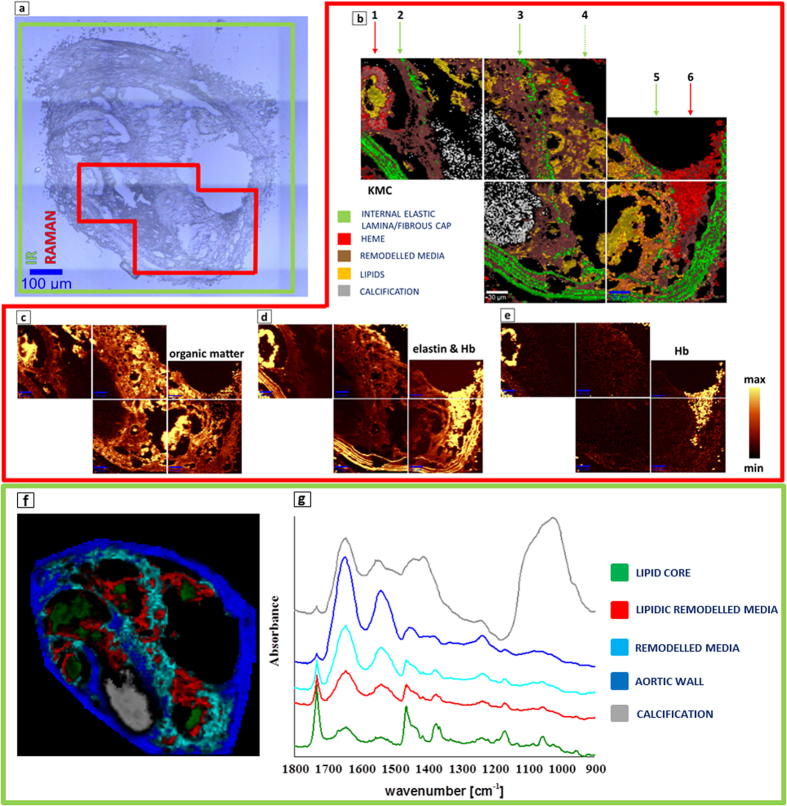
(**a**) A microphotograph of the cross section of a BCA taken from a 6-month-old ApoE/LDLR^−/−^ mouse fed LCHP diet with the labelled regions investigated with the use of IR (green) and Raman (red) areas; (**b**) The K-means Clustering (KMC) results with the 4 main classes including remodelled media, heme, internal elastic lamina/fibrous cap and lipids. Their average spectra are presented in Fig. 4; (**c**) Raman integration maps of a CH stretching band approx. in the 2800–3050 cm^−1^ region; (**d**) Autofluorescence of the sample connected with the presence of elastin features (internal elastic lamina and fibrous cap) and Hb; (**e**) Raman integration maps of a band centered at 1130 cm^−1^ or 745 cm^−1^ (Hb); The yellow color corresponds to the highest relative intensity of integrated band or distribution of compound/group of compounds. For Raman mapping the sampling density was equal to 1.67 μm. Color coding for the classes is presented in the figure; (**f**) A Fuzzy C-Means (FCM) clustering map of the whole section, based on FT-IR imaging data, with 5 classes. (**g**) The corresponding average spectra from FCM analysis – spectra were offset for clarity.

**Figure 4:**
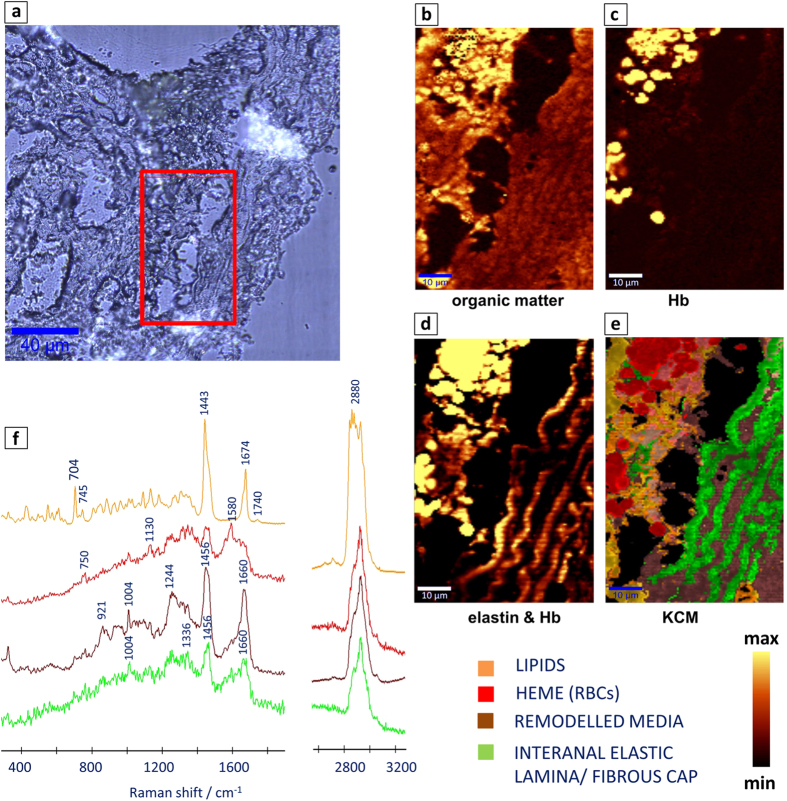
High resolution Raman measurements of a subset of the cross section of the BCA presented in Fig. 3. (**a**) A microphotograph of the intraplaque haemorrhage; (b) Raman integration maps of a CH stretching band approx. in the region 2800–3050 cm^−1^ and (**c**) a band centered at 1130 cm^−^1 or 745 cm^−^1 (Hb); (**d**) Autofluorescence of the sample connected with the presence of internal elastic lamina and Hb; The yellow color corresponds to the highest relative intensity of integrated band or distribution of compound/group of compounds; (**e**) The K-means Clustering (KMC) results with the 4 main classes including remodelled media, heme, internal elastic lamina and lipids with (**f**) the average spectra of the respective 4 classes. Color coding for the classes is presented in the figure; Sampling densities were equal to 500 nm.

**Figure 5:**
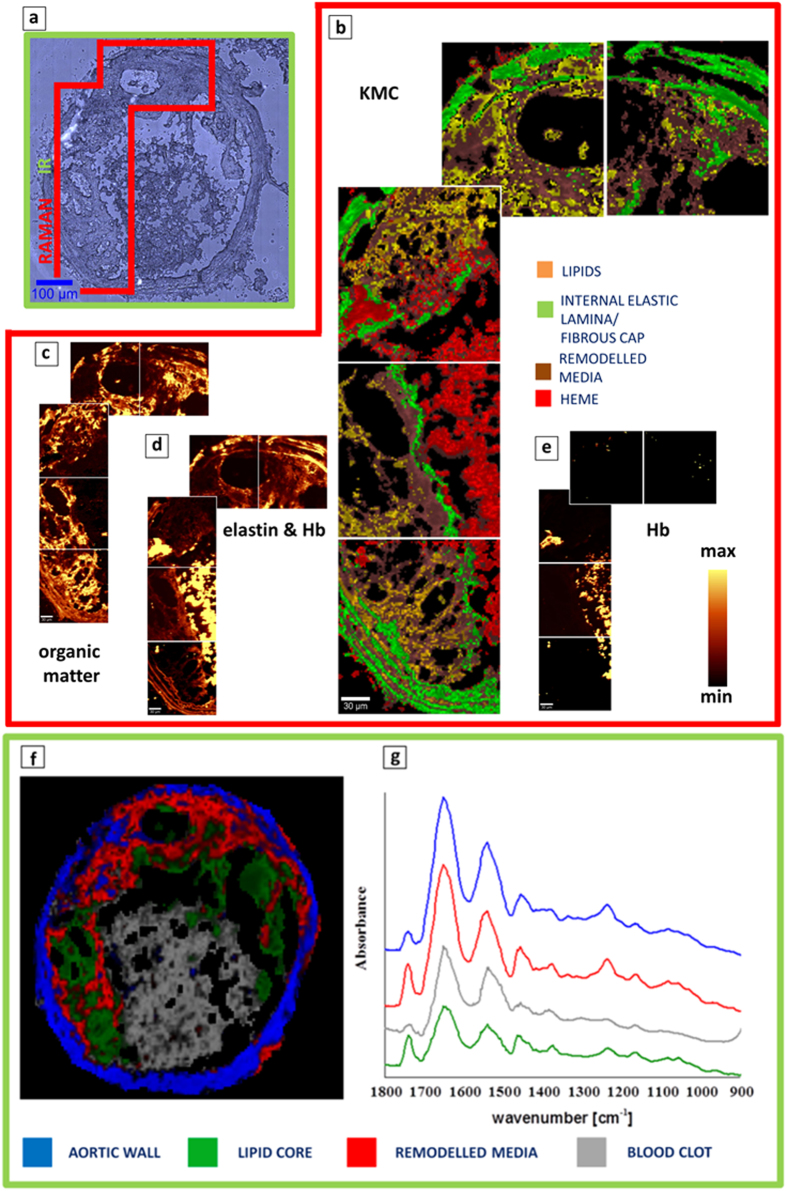
(**a**) A microphotograph of the cross section of a BCA taken from a 6-month-old ApoE/LDLR^−/−^ mouse fed AIN (control) diet with the labelled regions investigated with the use of IR (green) and Raman (red) areas; (**b**) The K-means Clustering (KMC) results with the 4 main classes including remodelled media, heme, internal elastic lamina/fibrous cap and lipids. Color coding for the classes is presented in the figure; (**c**) Raman integration maps of a CH stretching band approx. in the region 2800–3050 cm^–1^; (**d**) Autofluorescence of the sample connected with the presence of elastin features (internal elastic lamina and fibrous cap) and Hb; (**e**) Raman integration maps of a band centered at 1130 cm^−1^ or 745 cm^−1^ (Hb); For Raman mapping the sampling density was equal to 1.67 μm; (**f**) A FCM clustering map of the whole section, based on FT-IR imaging data, with 4 classes; (**g**) The corresponding average spectra from FCM analysis – spectra were offset for clarity.

Figures 3 and 4 present the representative cross sections of the BCA (ApoE/LDLR^−/−^ mouse fed LCHP diet) investigated with the use of IR and Raman imaging. More examples can be found in the Supplementary Materials and in our previous work comparing clustering results with histologic staining^8^,^10^. The presence of the lipid core inside of the atherosclerotic plaque of BCA is evidenced by bands connected with the cholesterol fatty acid esters and cholesterol. This class is presented in green color for FT-IR images and in yellow for Raman results (Figs 3, 4, 5). The average spectra of the lipid class are presented in Fig. 3g for FT-IR and Fig. 4f for Raman. The most prominent Raman bands can be observed at 1674 cm^−1^ (C = C stretching), 1443 cm^−1^ (C-H bending), 1740 cm^−1^ (C = O stretching), 2885 cm^−1^ at (C-H stretching) and at lower frequencies (704 cm^−1^) due to vibration modes of steroid rings. The IR signatures are quite similar with strong bands at 1735 cm^−1^ (C = O stretching), 2800–3025 cm^−1^ (CH_2_/CH_3_ stretching), 1470 cm^−1^ (C-H bending), 1170 cm^−1^ (C-O-C stretching) and a band characteristic for pure cholesterol at 1055 cm^−1^.

The band at 965 cm^−1^ is characteristic for calcified plaque and is assigned to the symmetric stretching vibration of phosphate groups in Raman, while in IR a very characteristic broad band in the range of 1200–1000 cm^−1^ is observed - white/gray class in Fig. 3. The internal and external elastic laminas contain mainly elastin and collagen. In all figures containing Raman maps, elastic lamina and fibrous cap are illustrated as a green class. These structures demonstrate weak Raman signal and strong autofluorescence when 532 nm laser excitation wavelength is used^10^. Remodelled tunica media (Raman brown class, Figs 3, 4, 5) corresponds to the pathologically altered muscle cells. Its Raman spectrum originates mainly from the protein features and the most prominent bands are observed at 1660 cm^−1^ (amide I), 1244 cm^−1^ (amide III) and at 1004 cm^−1^ (phenylalanine). IR spectroscopy is less specific with regard to aortic wall (blue class), since the Amide bands coming from proteins are not as straightforward to assign as Raman signatures. The observed hemoglobin distribution (Raman red class, Figs 3, 4, 5) may be ascribed to increased vulnerability of the plaque and intraplaque haemorrhages, or rather, as we suppose, to artifactual plaque rupture upon sample preparation. The average Raman spectrum of the heme class obtained with the use of 532 nm laser wavelength is observed at 745, 1130 and 1580 cm^−1^.

This cross section shows an interesting distribution of fibrous cap class which was observed in most of the plaques from mice fed LCHP diet and measured with the use of Raman spectroscopy. The fibrous cap normally surrounds the large lipid core while the K-Means Cluster analysis presented in Fig. 3 reveals existence of 3 buried fibrous caps marked with the green arrows 2, 3 and 5 from the middle to the external part of the plaque, respectively, that were not observed in plaques from mice fed AIN diet (Fig. 5). As we previously reported with the use of AFM technique and Raman spectroscopy^10^, the measured fibrous cap thickness for AIN plaques varied in the range of 6–11 micrometers. Here, with the use of Raman spectroscopy (width of the fibrous cap pixels obtained with the use of KCM analysis) it was observed that the relative thickness of the outer fibrous cap decreased as compared to fibrous caps of mice fed AIN diet. Fibrous caps of LCHP plaque were never wider than 8–9 micrometers and in many places were also interrupted. More examples may be found in the Supplementary Materials (Figure S2). The FT-IR data of the same section are presented in Fig. 3f,g, with 3f showing the Fuzzy C-Means (FCM) clustering. The gray class in Fig. 3g is clearly identified as calcification. The remaining 4 classes represent a changing proteins:lipids ratio and span a range from the most protein-rich class of aortic wall (IR blue), through remodelled (IR cyan) and lipidic remodelled (IR red) media up to the lipid core (IR green). The lipid core had an extremely high content of fats with very little proteins – the ratio being higher than in AIN sections as seen in Fig. 5.

## Discussion

Low Carbohydrate High Protein diets are very popular worldwide. These diets have been proposed as conducive to weight loss. However, little is known about these diets’ long-term effects on vascular health. Foo *et al.*^15^ provided initial insight into this area, demonstrating that mice fed LCHP diet for 12 weeks showed a significant increase in atherosclerosis. Kostogrys *et al.*^14^ also confirmed that the LCHP diet significantly increased the extent of advanced atherosclerosis in the ApoE/LDLR^−/−^ mice both in aortic root and in BCA (the proatherogenic effect was even more extensive than in Western Diet fed mice).

In the present work with the use of FT-IR and Raman imaging, we characterized biochemical content of plaques in BCA from ApoE/LDLR^−/−^ mice fed LCHP diet as compared to AIN diet. The LCHP diet induced significant changes in the lipid fraction of the atherosclerotic plaque. We found that in aortic plaques from ApoE/LDLR^−/−^ mice fed LCHP diet, the content of cholesterol and cholesterol esters was increased, while that of proteins decreased as evidenced by FT-IR analysis. Interestingly, biochemical FT-IR-based plaque imaging detected areas of very low protein content and high cholesterol ester content.

Van de Poll *et al.*^17^ demonstrated that Raman spectroscopy can be applied to the study of atherosclerotic plaque development in terms of plaque area and composition. By calculating the relative contributions of cholesterol, calcification and protein derived from the arterial tissue, Raman spectra maps reflecting the distribution of components in the aortic arch of mice were generated. Most studies on atherosclerosis in the aorta have focused on the aortic roots or ascending aorta including the aortic arch^17^. In this study atherosclerotic plaque analysis was focused on BCA lesions closely resembling those in human atherosclerosis. Our previous work clearly suggest that BCA’s are vessels of choice to reliably analyze efficacy and mechanisms of anti-atherosclerotic treatment in ApoE/LDLR^−/−^ mice^16^.

In the present work increased cholesterol and particularly cholesterol esters content was observed in plaques from ApoE/LDLR^−/−^ mice fed LCHP diet. Cholesterol esters are a major fraction of the lipid-rich core of the plaque, and their abundance has been linked with plaque rupture and formation of atherothrombosis^18^. Moreover, in the current study decreased protein content in the atherosclerotic plaque in LCHP fed mice was observed. Since collagen plays a key role in determining plaque’s stability^19^ these results indicate that the LCHP diet, paradoxically by diminishing protein content, can lead to instability of the atherosclerotic plaque in mice.

The plaques of LCHP fed mice were rich in buried fibrous caps, which were not observed for AIN fed mice and the fibrous cap tend to be thinner in LCHP fed mice. Furthermore, the protein content and composition of the lipid core of the plaque has been considered to play a major role in vulnerability of the plaque^18^. Van de Poll *et al.*^20^ has shown that accumulation of noncrystalline cholesteryl esters may soften the lipid core and results in a thin, fibrous cap, which is more prone to rupture. Our results support the occurrence of thin fibrous cap in LCHP-fed mice, but obviously further studies using AFM or other methods are necessary to support our findings with quantitative analysis. Nevertheless, LCHP diet increased plaque cholesterol and cholesterol esters contents of atherosclerotic plaque, supporting the cholesterol-driven pathogenesis of LCHP–induced atherogenesis in ApoE/LDLR^−/−^ mice.

## Conclusions

In atherosclerotic plaques from LCHP diet fed ApoE/LDLR^−/−^ mice the content of cholesterol and cholesterol esters was increased, while that of proteins was decreased as evidenced by FT-IR analysis. Biochemical FT-IR-based plaque imaging detected areas of low protein and high cholesterol ester content. In turn, high resolution imaging by RS identified necrotic core/foam cells, lipids (including cholesterol crystals), calcium mineralization and a fibrous cap. Decreased relative thickness of the outer fibrous cap and the presence of buried caps was a prominent feature of the plaques in ApoE/LDLR^−/−^ mice fed LCHP diet. FT-IR and Raman-based imaging provided a complementary insight into the biochemical composition of the plaque suggesting that LCHP diet increased plaque cholesterol and cholesterol esters contents of atherosclerotic plaque, supporting the cholesterol-driven pathogenesis of LCHP–induced atherogenesis.

## Materials and Methods

### Tissue preparation

All procedures involving animals were conducted in accordance with the Guidelines for Animal Care and Treatment of the European Union and were approved by the 1^st^ Local Animal Ethics Commission in Krakow (decision no. 51/2009).

The Brachiocephalic artery (BCA) were taken from ApoE/LDLR^−/−^ mice fed LCHP diet for 2 months, up to 6 month old. The experimental approach was adapted from a previous study^14^ as optimal for atherosclerotic plaque development. The LCHP diet contained corn starch −5%; caseine −52%; butter – 21%. Standard AIN-93G diet, used as a control, contained corn starch −53%; caseine −20%; soybean oil – 7%. The amount of mineral mix and vitamin mix in both experimental diets was 3.5% and 1%, respectively.

Isolated BCA’s were fixed for 6 hours in 4% buffered formalin. After fixation, BCA were mounted for the next 6 hours in a tissue freezing medium (Leica, Germany) with distilled water (1:1) and finally embedded in the OCT medium and frozen at −80 °C. The 5 μm cross section sections were put on CaF_2_ slides. LCHP diet and AIN-93G diet groups consisted of 5 and 6 animals, respectively, with 6–10 sections per animal giving the final dataset equal to 41 (LCHP) and 53 (AIN) sections.

### FT-IR data acquisition and analysis

FT-IR imaging experiments were performed using the Varian 620-IR microscope coupled to a 670-IR spectrometer with a liquid nitrogen cooled mercury-cadmium-telluride (MCT) and 128 × 128 pixel focal plane array (FPA) detector. Measurements were obtained in transmission mode and the acquired maps were always a multiple of 128 × 128 pixels with a FOV of 700 × 700 μm^2^ – in most cases only 1 tile was needed. For background and sample measurements 128 and 32 scans were collected. The total acquisition time was approximately 5–10 min for each image. All spectra were obtained with the spectral resolution of 4 cm^−1^. Clustering and part of preprocessing of the collected images was performed using CytoSpec software (ver. 2.00.01). Final data managing and visualization was done in MatLab 10 environment (The MathWorks, Natick, MA) with in-home built scripts.

The preprocessing stage consisted of spatial and chemical filtering. Spatial filtering was done using Region of Interest (ROI) function implemented in Cytospec and was performed to discard adventitia tissue, which covers blood vessels and has very high concentration of lipids (which signal would be confused with the lipids present in the plaque). The oval of the blood vessel was marked by hand by the user for each of the images. Afterwards, a cutoff value based on integration in the 2800–3025 cm^−1^ range was used, in order to discard areas without tissue – the effect of such preprocessing can be seen in Figure S1. The images were then subjected to band integration in selected 22 ranges, corresponding to known bands.

The global chemical composition changes are a good measure of the central tendency, however, very often changes occur also in the spatial rearrangement of the constituents^13^. A way of observing such changes is to investigate integration maps of bands corresponding to different chemicals. However, this approach is time consuming and subjective, therefore, it is proposed to use scatter plots of individual pixel’s integration, to have a fast insight into the colocalization of the chemical species.

#### Statistical analysis

For each of the ranges and for each of the images, a median was taken, as a measure of a distribution in that image and range. Next, the medians from all LCHP images were compared to medians from all control images by means of Mann-Whitney-Wilcoxon (MWW) test for equality of distributions.

### Raman data acquisition and analysis

Raman imaging of BCA cross sections was performed on a WITec confocal CRM Alpha 300 Raman microscope. The spectrometer was equipped with an air–cooled solid state laser operating at 532 nm and a CCD detector, which was cooled to –65 °C. The laser was coupled to a microscope via a single mode optical fiber with a diameter of 50 μm. The scattered radiation was focused onto a multi–mode fiber (50 μm diameter) and a monochromator. The integration time for a single spectrum was 0.3 s with a spectral resolution of 3 cm^−1^. The monochromator of the spectrometer was calibrated using the Raman scattering line produced by a silicon plate (520.7 cm^−1^). For Raman map collection a water immersive Nikon (×60/1NA) objective was used. The power for all measurements of the laser at the sample position was ≤10 mW. Such power allowed us to measure cross sections slides of tissues without photo-damage of any types of measured cells.

Raman data analysis was performed with Opus^TM^ and WITec software^TM^. Raman maps were generated based on the integration of marker bands and were obtained without pre–processing. Cluster analysis was carried out after cosmic ray spike removal and background subtraction. K–Means Clustering (KMC) results were obtained with the Manhattan distance algorithm and are complementary to the analysis based on the integration of the specific marker bands.

## Additional Information

**How to cite this article**: Wrobel, T. P. *et al.* Effects of Low Carbohydrate High Protein (LCHP) diet on atherosclerotic plaque phenotype in ApoE/LDLR^−/−^ mice: FT-IR and Raman imaging. *Sci. Rep.* **5**, 14002; doi: 10.1038/srep14002 (2015).

## Supplementary Material

Supplementary Information
