# Supplementary material for: Effects of Low Carbohydrate High Protein (LCHP) diet on atherosclerotic plaque phenotype in ApoE/LDLR−/− mice: FT-IR and Raman imaging
Source: Sci Rep. 2015 Sep 22;5:14002. doi: 10.1038/srep14002 (PMC4585723; doi:10.1038/srep14002)
Supplement: Supplementary Information [file srep14002-s1.pdf]

Supplementary Materials

Effects of Low Carbohydrate High Protein (LCHP) diet on atherosclerotic plaque phenotype in ApoE/LDLR$^{-/-}$ mice: FT-IR and Raman imaging

T.P. Wrobel, K.M. Marzec, S. Chlopicki, E. Maślak, A. Jasztal, M. Franczyk-Żarów, I. Czyżyńska-Cichoń, T. Moszkowski, R.B. Kostogrys, and M. Baranska

Quantification of chemical components based on FT-IR images and spectra

The preprocessing stage consisted of spatial and chemical filtering. Spatial filtering was done using Region of Interest (ROI) function implemented in Cytospec and was performed to discard adventitia tissue, which covers blood vessels and has very high concentration of lipids (which signal would be confused with the lipids present in the plaque). The oval of the blood vessel was marked by hand by the user for each of the images. Afterwards, a cutoff value based on integration in the 2800-3025 cm^{-1} range was used, in order to discard empty pixels – the effect of such preprocessing can be seen in Figure S1. The images were the subjected to integration in 22 ranges, corresponding to the most prominent bands. For each of the ranges and for each of the images, a median was taken, as an estimate of a distribution in that image and range. A median was chosen over a mean, since all of the distributions of the integrated ranges within a single image were skewed. The non-parametric Kolmogorov–Smirnov test for normality of distributions was performed – none of the distributions passed within $p<0.05$. Next, the medians from all LCHP images were compared to medians from all AIN images by means of Mann-Whitney-Wilcoxon (MWW) test for equality of distributions.

Figures

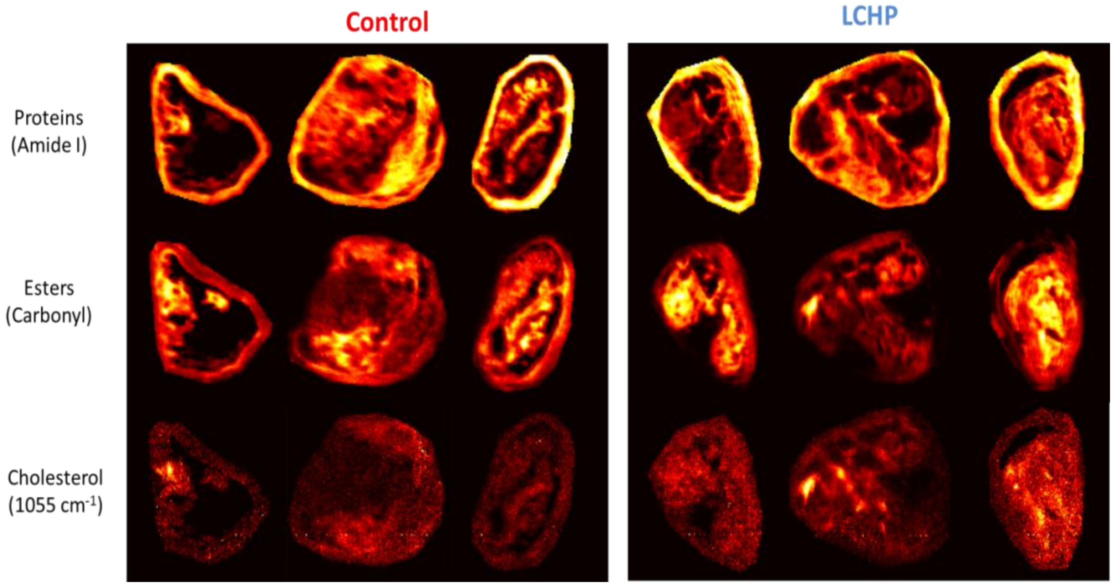

Figure S1. Exemplary integration maps of proteins, esters and cholesterol bands for 3 control and 3 LCHP sections. For a given integration, the intensity scales are the same with white being the maximum and dark brown the minimum.

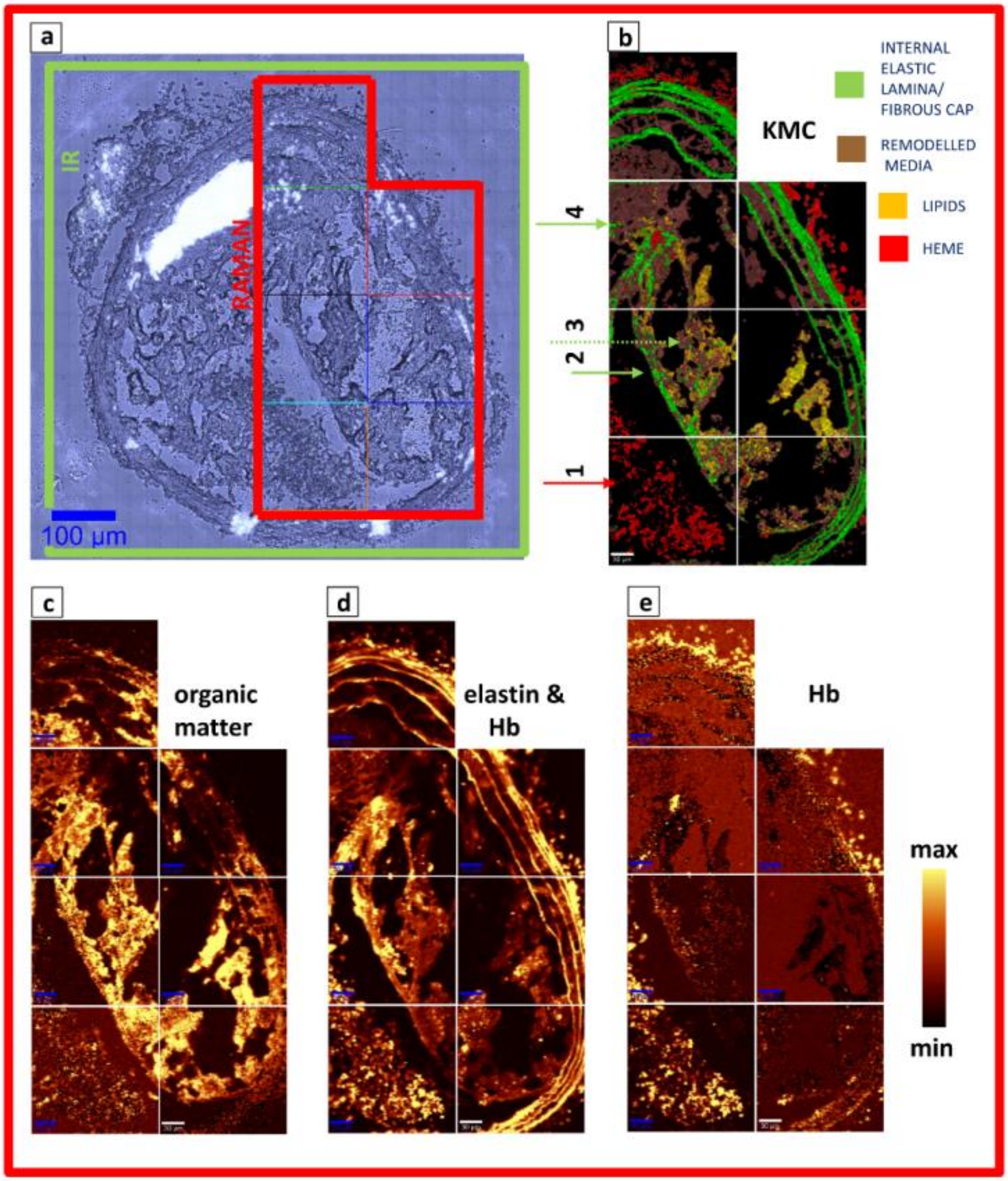

Figure S2. (a) A microphotograph of the cross section of a brachiocephalic artery taken from a 6-month-old ApoE/LDLR$^{-/-}$ mouse fed with LCHP diet with the labelled regions investigated with the use of IR (green) and Raman (red) areas; (b) The K-means Clustering (KMC) results with the 4 main classes including remodeled media, heme, internal elastic lamina/fibrous cap and lipids. The average spectra of the respective 4 classes are present in Figure 4. Color coding for the classes presented in the figure is presented; (c) Raman integration maps of a CH stretching band approx. in the region 2800–3050 cm^{-}1,(d) Autofluorescence of the sample connected with the presence of elastin features (internal elastic lamina and fibrous cap) and Hb; (e) Raman integration maps of a band centered at 1130 cm^{-}1 or 745 cm^{-}1 (Hb); The yellow color

corresponds to the highest relative intensity of integrated band or distribution of compound/group of compounds. Sampling densities were equal to 1.67 µm.

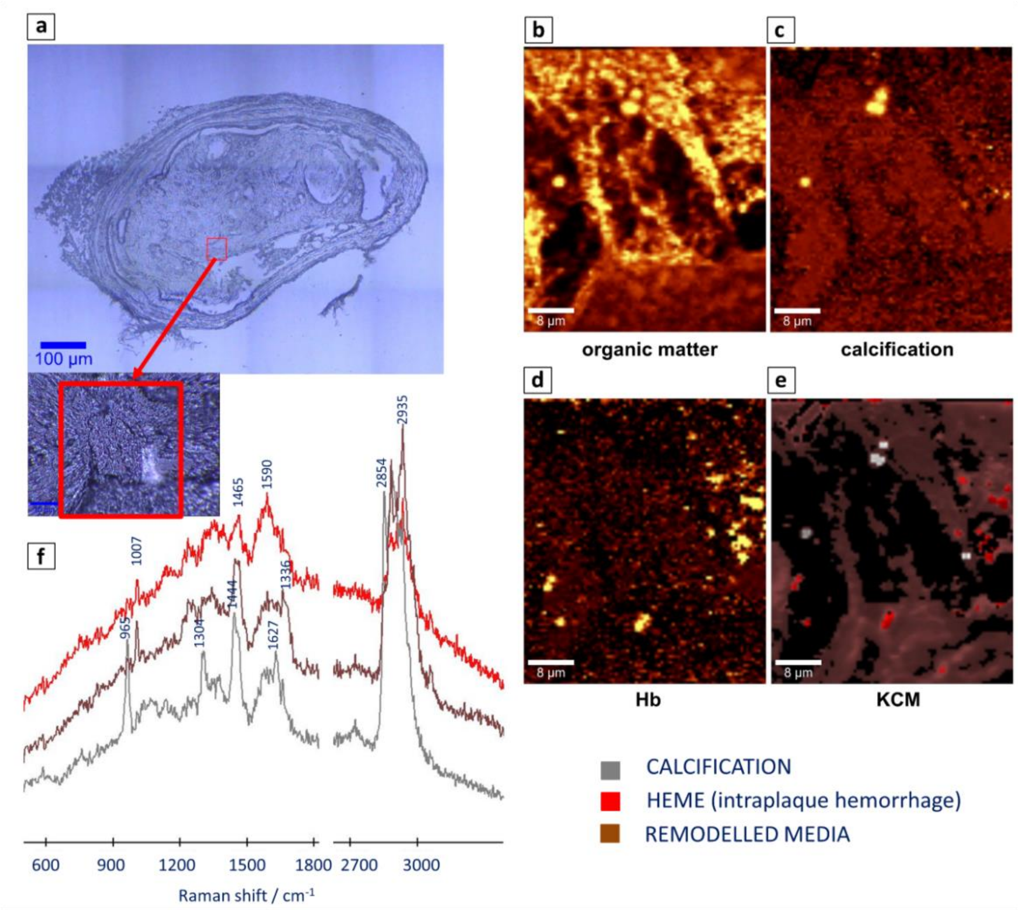

Figure S3. (a) A microphotograph of the cross section of a brachiocephalic artery taken from a 6-month-old ApoE/LDLR$^{-/-}$ mouse fed with LCHP diet with the labelled regions investigated area with the use of Raman (red); Raman integration maps of (b) a CH stretching band approx. in the region 2800–3050 cm^{-}1, (c) the symmetric stretching vibration of phosphate groups at 965 cm^{-1} and (d) band centered at 1590 cm^{-}1 connected with red blood cells presence (Hb). The yellow color corresponds to the highest relative intensity of integrated band or distribution of compound/group of compounds. (e) The K-means Clustering (KMC) results with the 3 main classes including remodeled media, heme, internal elastic lamina/fibrous cap and lipids with (f) the average Raman spectra. Color coding for the classes presented in the figure is presented; Sampling densities were equal to 500 nm.

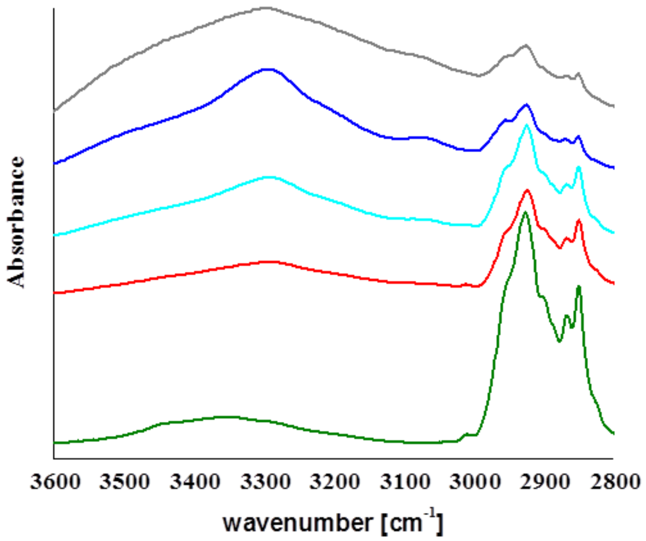

Figure S4. High wavenumber range of mean FCM class spectra, corresponding to results in Figure 3.
